# A small stretch of poor codon usage at the beginning of dengue virus open reading frame may act as a translational checkpoint

**DOI:** 10.1186/s13104-023-06615-5

**Published:** 2023-12-05

**Authors:** Maneenop Yimyaem, Kunlakanya Jitobaom, Prasert Auewarakul

**Affiliations:** 1 Graduate Program in Molecular Medicine, Faculty of Science, Mahidol University, Princess Srisavangavadhana College of Medicine, Chulabhorn Royal Academy, Bangkok, Thailand; 2 Department of Microbiology, Faculty of Medicine Siriraj Hospital, Mahidol University, Bangkok, Thailand

**Keywords:** Rare codon, Codon adaptation index, Translational checkpoint

## Abstract

**Objective:**

Rare codons were previously shown to be enriched at the beginning of the dengue virus (DENV) open reading frame. However, the role of rare codons in regulating translation efficiency and replication of DENV remains unclear. The present study aims to clarify the significance of rare codon usage at the beginning of DENV transcripts using the codon adaptation index (CAI).

**Methodology:**

CAIs of the whole starting regions of DENV transcripts as well as 18-codon sliding windows of the regions were analyzed.

**Results:**

One of the intriguing findings is that those rare codons do not typically result in uniformly low CAI in the starting region with rare codons. However, it shows a notable local drop in CAI around the 50th codon in all dengue serotypes. This suggests that there may be a translational checkpoint at this site and that the rare codon usage upstream to this checkpoint may not be related to translational control.

**Supplementary Information:**

The online version contains supplementary material available at 10.1186/s13104-023-06615-5.

## Introduction

In the standard genetic code table, 61 codons correspond to the 20 amino acids. Even though synonymous codons encode the same amino acid, their distribution in the genomes of all organisms is not random causing codon usage bias [1].

Codon usage bias differs among genomes of species including viruses [1, 2], and can differ even among gene groups within the same genome [3, 4]. However, some genes may show little or no codon usage bias [5]. Most viruses have codon usage pattern unrelated to their host [6, 7]. Viral codon usage may depend on their genome composition and the abundance of tRNA [8]. Codon usage may affect gene expression at both the transcriptional and translational levels [2]. Optimal codons match with abundant tRNAs and properly interact with anticodons leading to efficient translation [9]. In contrast, the use of rare codons results in a decrease in translation rate [10].

Rare codons can be found in the genomes of a wide range of organisms [11] and are also common in viruses. Previous studies showed that rare codons are over-represented in the initiation site of DENV and West Nile virus mRNA [12, 13], but not in the hepatitis C virus [14]. It was proposed that these rare codons may provide a translational regulation [12, 13].

Tuller et al. [15]. previously showed that the first 30–50 codons of genes are translated with a low efficiency corresponding to low abundant tRNAs by measuring the tRNA adaptation index (tAI). They proposed the “ribosomal traffic rules” so that the rare codons serve as a ramp, in which ribosomes pause and form a queue thereby preventing ribosome collisions. Here, we attempt to understand the role of rare codons at the beginning of the DENV open reading frame by investigating their CAI.

## Materials and methods

### DENV genome sequence data

A total of 160 strains of the four DENV serotypes (DENV1-4) were used in this study (Additional file 1: Table S1). We randomly selected these strains from geographically isolated regions of the world, such as Africa, Asia, Europe, North America, South America, the Caribbean, and the Pacific. The datasets for the strain, isolated region, year of isolation, genome length, and GenBank accession number are also shown in this table. The complete DENV genome sequences were obtained from the National Center for Biotechnology Information (NCBI) GenBank database.

### Relative synonymous codon usage analysis

The relative synonymous codon usage (RSCU) analysis is used to measure the degree of codon usage bias for each codon of each amino acid in a coding sequence. To characterize the synonymous codon usage bias in the various initiation sites of the four DENV serotype coding sequences, the first 25, 50, 75, and 100 codon sites, as well as the entire DENV genome, were prepared as follows: A total of 40 strains of each serotype were excised at the indicated individual codon positions from the start codon, followed by assembly of each serotype strain using BioEdit version 7.2.5. The RSCU values of each codon were calculated using the automated codon usage analysis software (ACUA version 1.0), which can be downloaded from http://www.bioinsilico.com/acua [16]. The synonymous codons with RSCU values > 1.6 and < 0.6 were over- and under-represented codons, respectively, while codons with RSCU values between 0.6 and 1.6 are considered unbiased or randomly used [3, 14, 17–19]. The RSCU values of human highly-expressed genes were calculated using the following formula:

$${\text{RSCU}}_i= \frac{Xi}{\frac{1}{n}\sum\limits _{i=1}^{n}Xi}$$

where _*Xi*_ is the number of occurrences of synonymous codon *i*, and *n* is the number of synonymous codons for that amino acid [9].

### Codon adaptation index analysis

The codon adaptation index (CAI) analysis was used to measure synonymous codon usage bias in a coding sequence, gene expression level, and translation efficiency [20, 21]. The CAI value of a coding sequence was calculated using the CAIcal (http://ppuigbo.me/programs/CAIcal/), which required a reference set of known highly expressed genes [22]. A CAI value ranges from 0 to 1.0, with a higher value indicating a stronger codon usage bias as well as a higher degree of translation efficiency. In the present study, CAI analysis of the initiation site along the four DENV serotype coding sequences was calculated using codon usage reference tables for *Homo sapiens* and *Aedes aegypti* (Additional file 2: Table S2) from the codon usage database (http://www.kazusa.or.jp/codon/). A sliding window of CAI output along the gene sequence was assigned using a window size of 18 codons and a window step of 5 codons.

## Results

### Rare codon usage at the 5’ region of DENV mRNA

**Fig. 1:**
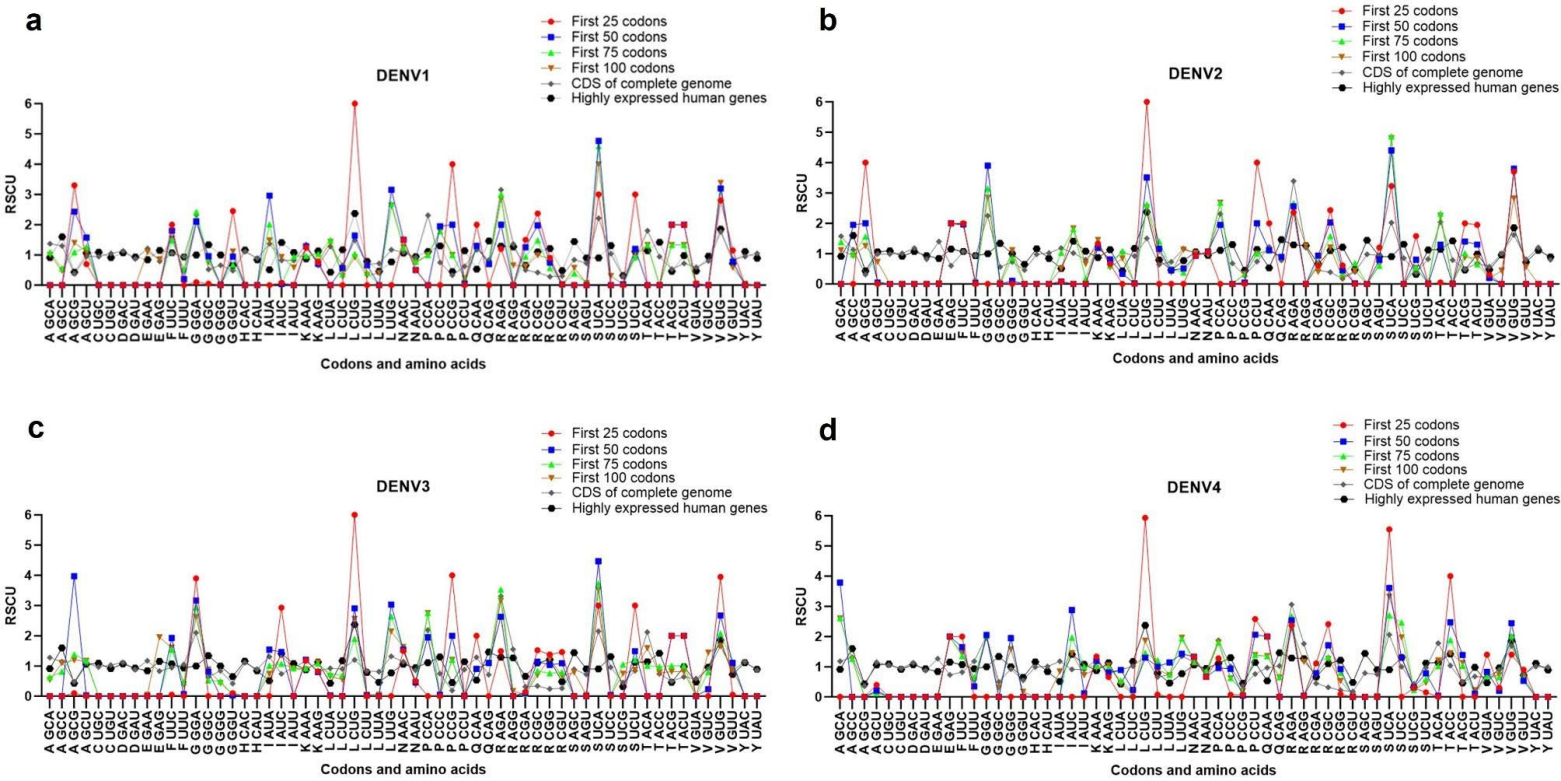
RSCU values of each codon in the initiation sites of DENV1-4. The RSCU values of DENV1 (**a**), DENV2 (**b**), DENV3 (**c**), and DENV4 (**d**), as well as RSCUs of human highly-expressed genes are represented by the y-axis. The codon families for each amino acid are provided on the x-axis. Data in Additional file 3: Table S3 is presented as line graphs

We calculated the RSCU values to determine the synonymous codon usage pattern in the various initiation sites of the four DENV serotypes coding sequence, the first 25, 50, 75, and 100 codon sites, as well as the entire DENV genome, as shown in Fig. 1 (graphical-based representation) and Additional file 3: Table S3 (calculated RSCU data). The zero RSCU values were caused by unused or absent synonymous codons in those sequences. Among the 59 synonymous codons in the DENV1-4 coding sequence, six were observed to be over-represented: GGA for Gly, CCA for Pro, AGA for Arg, UCA for Ser, ACA for Thr, and GUG for Val, and nine were found to be under-represented: GCG for Ala, GGU for Gly, CCG for Pro, CGA, CGC, CGG, and CGU for Arg, UCG for Serine, and ACG for Thr. The most frequently used codon in DENV1-4 genomes was AGA for Arg. The codon usage of the 5’regions appeared to be different from the usage pattern of the whole genome. The CUG for Leu, AGA for Arg, UCA for Ser, and GUG for Val codons were persistently over-represented in all the 5’ regions with various lengths, whereas many under-represented codons in the whole genomes such as CGC for Arg were more frequently used in the 5’ regions. Some of the over-represented codons in the 5’ regions such as GCG for Ala were rare codons in human codon usage. It was previously proposed that this different codon usage in the initiation region of DENV mRNA may affect translational efficiency and provide some translational control [12, 13].

To understand the role of this codon usage, we calculated CAIs of the whole genomes and the 5’regions. In accordance with previous reports [23], CAIs of DENV full length sequences were found to be relatively low (Additional file 4: Table S4). However, except for slightly lower CAIs at the first 25–75 codons in DENV1 and the first 75 codons of DENV2, the CAIs of the 5’regions were not significantly lower than those of the entire genome. Similarly, when compared to *Ades* mosquito vector codon usage, the CAIs of the 5’regions were not lower than those of the entire genome (Additional file 5: Table S5). This was unexpected and could be explained by the presence of some optimal codons such as CUG for Leu and GUG for Val in this region, making the CAI no lower than the average of the genome despite the overrepresentation of rare codons.

### A putative translational checkpoint was found around the 50th codon

**Fig. 2:**
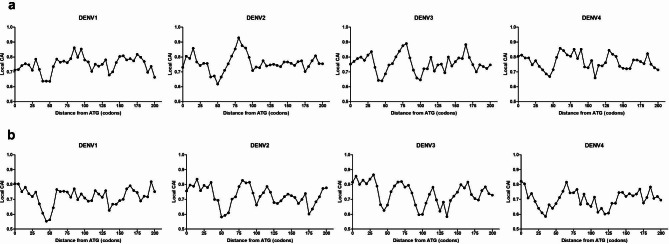
Local translation efficiency profiles in DENV1-4. CAI is calculated in the first 200 codons using various reference sets of codon usage: *Homo sapiens* (**a**) and *Aedes aegypti* (**b**), with a sliding window of 18-codon lengths across the codon sequence and a window step of 5 codons

To further investigate how the presence of rare codons in the initiation site of mRNA affects local translation efficiency of DENV in human and *Aedes* mosquito cells, CAI values of sliding 18-codon fragments were calculated in the first 200 codons across the codon sequence of four DENV serotypes using a reference set of highly expressed genes for *Homo sapiens* and *Aedes aegypti* (Additional file 6: Table S6 and Additional file 7: Table S7). As shown in Fig. 2, we found that the local CAI profiles gradually decreased from the start site, then uniformly dropped to the lowest CAI at the 50th -codon position. These results showed that the presence of rare codons at the beginning of DENV1-4 transcripts resulted in low CAI values at the 50th -codon specific position with a boundary of 38 codons, indicating a low level of mRNA translation and thereafter a slowing down of translation speed. The region with a local decline in CAI is proposed as a translational checkpoint with a local slow translation rate. Although there are some other local dips in the CAIs at some other positions, for example a dip at 100th codon for DENV3, the local drop at the 50th is the lowest and the only uniform one among all DENVs.

### Synonymous codon usage bias in the translational checkpoint of DENV

**Fig. 3:**
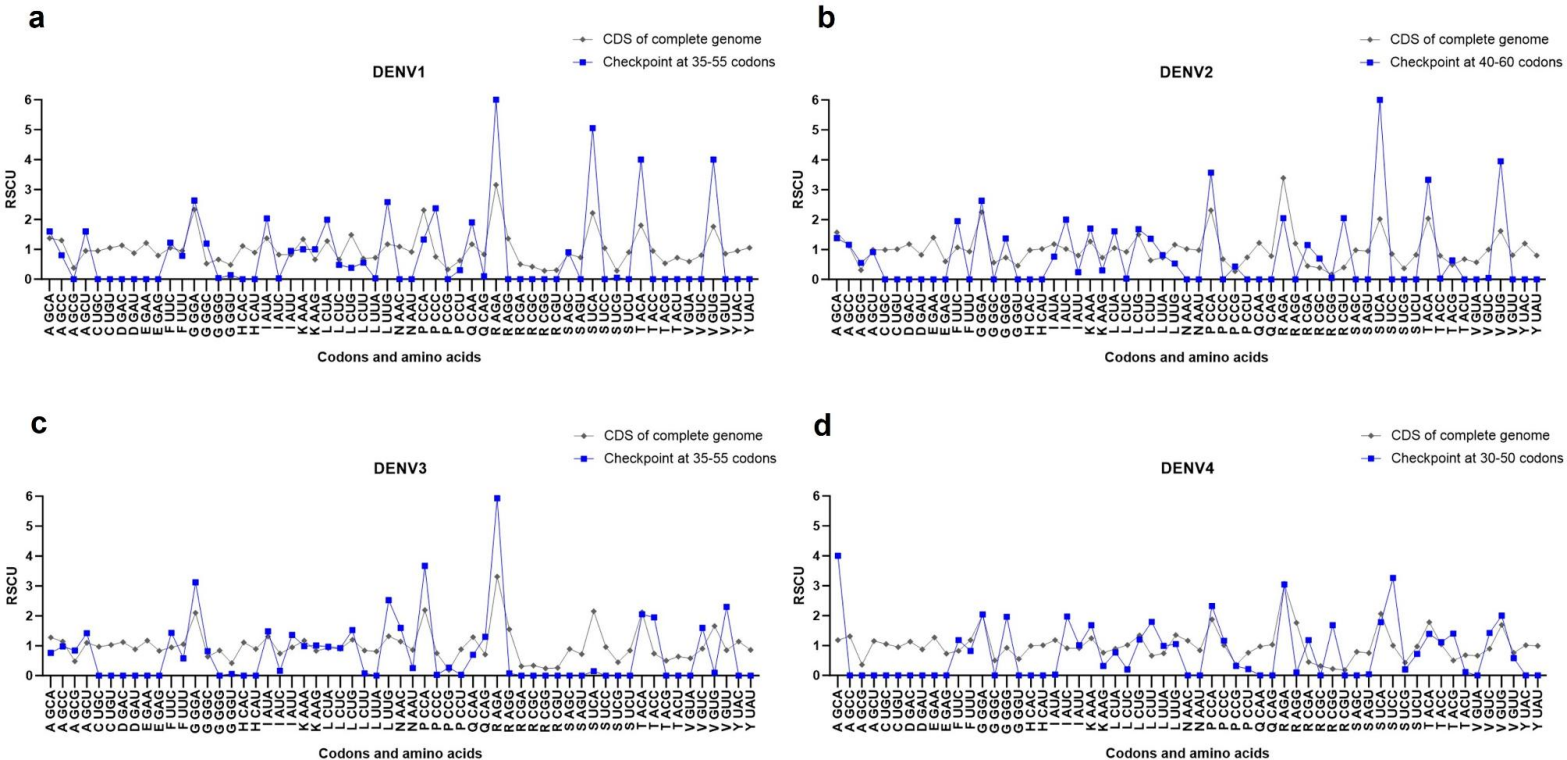
RSCU values of each codon in the translational checkpoint of DENV1-4. The RSCU values of DENV1 (**a**), DENV2 (**b**), DENV3 (**c**), and DENV4 (**d**) are represented by the y-axis. The codon families for each amino acid are provided on the x-axis

To analyze the synonymous codon usage bias pattern at the translational checkpoint of the four DENV serotypes, we calculated the RSCU values for the codon sequences at the translational checkpoint in DENV1-4 (Fig. 2a) as follows: checkpoint at 35–55 codons for DENV1, checkpoint at 40–60 codons for DENV2, checkpoint at 35–55 codons for DENV3, and checkpoint at 30–50 codons for DENV4. As indicated in Fig. 3 and Additional file 8: Table S8 (calculated RSCU data), each serotype of DENV showed the synonymous codon usage bias pattern in the translational checkpoint with notable over-represented codons (RSCU values > 1.6). These included AGA (Arg), UCA (Ser), ACA (Thr), and GUG (Val) for DENV1; UCA (Ser), ACA (Thr), and GUG (Val) for DENV2; AGA (Arg) for DENV3; and UCC (Ser) for DENV4. These codons are most frequently used in their codon usage tables, with the exception of UCC (Ser) for DENV4. However, most of these codons were not found in the codon usage pattern of the first 25 or 50 codon sites (Fig. 1), excluding UCA (Ser) and GUG (Val) for DENV1 and DENV2.

## Discussion

The over- and under-represented codons are consistent with the earlier studies [12, 24]. In their genome, all four DENV serotypes prefer A-ending codons, with the exception of GUG for Val. The codon AGA for Arg is the most preferred codon by DENV and other *Flaviviridae* family viruses, excluding hepatitis C virus [25]. In addition, codons containing CpG dinucleotides are under-represented in agreement with previous reports [24, 26].

The hypothesis proposed that the presence of rare codons at the beginning of mRNA transcripts may contribute to the slowing down of translation in order to maintain optimal protein expression levels, thereby increasing translation efficiency. Our result, on the other hand, suggests that the translation slowing down may not start from the beginning but only involve a narrow checkpoint around the 50th codon, and that the rare codons upstream to this checkpoint may be there for other reasons.

The codon adaptation index (CAI) is the primary usage for determining the efficiency of translation elongation rate in the coding region of all species [20, 27]. The results of CAI analysis along the first 200 codons of DENV coding sequences, given in Fig. 2 shows the local delay of the translational checkpoints in the 50th -codon region with a boundary of 38 codons in all four DENV serotypes. Our findings are supported by the mechanism of intragenic pattern of codon usage of Tuller *et al* [15].: the slow “ramp” of the presence of rare codons in the first 30–50 codons of the genes is translated with low efficiency and presumably reduces the ribosomal traffic jam, thereby preventing ribosome collisions during translation elongation, which can lead to mRNA degradation [28], and thus improving translation efficiency. Interestingly, the local CAI does not tend to decrease throughout the 50 codons at the 5’end of the transcript, implying that the translational checkpoint might not have to be 50 codons long starting at the 5’ end. This could be due to the difference between viral mechanisms and common host systems. It might be because the virus needs to eliminate constraints in the translation initiation region to enhance the efficiency of protein synthesis. As viruses require efficient viral protein expression, the translational checkpoint may be essential for viral replication and pathogenesis. Interestingly, the checkpoint was found at similar position in both the context of human and mosquito codon usage. This suggests that the checkpoint evolved for the adaptation to both human and mosquito hosts simultaneously. Elimination of the checkpoint may provide a new approach for viral attenuation. As rare codons were also found at the 5’ region of other flaviviruses, the checkpoint mechanism may also be present in other flaviviruses. Whether similar checkpoints are also present in other types of viruses requires further studies.

Altogether, our results suggest that the presence of local low CAI checkpoints around the 50th codon region of DENV sequences may provide a translational regulation. This supports the notion that the translation elongation speed may be regulated by the codon usage pattern.

## Limitations

We analyzed a limited number of DENV strains. However, these strains were randomly selected from geographically isolated regions of the world, such as Africa, Asia, Europe, North America, South America, the Caribbean, and the Pacific. Moreover, the complete genome sequences of DENV strains revealed little variation in codon usage and nucleotide composition.

### Electronic supplementary material

Below is the link to the electronic supplementary material.

Additional file 1: Table S1. Information of DENV complete genomes

Additional file 2: Table S2. The codon usage table of organisms

Additional file 3: Table S3. RSCU values of DENV1-4 in the CDS of the entire genome and their various initiation sites; the first 25, 50, 75, and 100 codons and the host

Additional file 4: Table S4. The CAI data of DENV1-4 in the CDS of the entire genome and their various initiation sites; the first 25, 50, 75, and 100 codons using codon usage table of Homo sapiens as a reference set

Additional file 5: Table S5. The CAI data of DENV1-4 in the CDS of the entire genome and their various initiation sites; the first 25, 50, 75, and 100 codons using codon usage table of Aedes aegypti as a reference set

Additional file 6: Table S6. Local CAI of DENV1-4 using codon usage table of Homo sapiens as a reference set

Additional file 7: Table S7. Local CAI of DENV1-4 using codon usage table of Aedes aegypti as a reference set

Additional file 8: Table S8. RSCU values of checkpoint for DENV1-4

## Data Availability

The datasets generated in this study are available in the manuscript and additional files.

## Abbreviations

ACUA: Automated Codon Usage Analysis
CAI: Codon Adaptation Index
CDS: Coding sequence
DENV: Dengue virus
mRNA: Messenger RNA
NCBI: Center for Biotechnology Information
RSCU: Relative Synonymous Codon Usage
tAI: tRNA Adaptation Index
tRNA: Transfer RNA
