# Supplementary material for: A small stretch of poor codon usage at the beginning of dengue virus open reading frame may act as a translational checkpoint
Source: BMC Res Notes. 2023 Dec 5;16:359. doi: 10.1186/s13104-023-06615-5 (PMC10698908; doi:10.1186/s13104-023-06615-5)
Supplement: Supplementary file 5 — Additional file 5: Table S5. The CAI data of DENV1-4 in the CDS of the entire genome and their various initiation sites; the first 25, 50, 75, and 100 codons using codon usage table of Aedes aegypti as a reference set [file 13104_2023_6615_MOESM5_ESM.pdf]

Table S2. The codon usage table of organisms.

The codon usage table of *Homo sapiens*.

Homo sapiens [gbpri]: 93487 CDS's (40662582 codons)

fields: [triplet] [frequency: per thousand] ([number])

UUU 17.6(714298)	UCU 15.2(618711)	UAU 12.2(495699)	UGU 10.6(430311)
UUC 20.3(824692)	UCC 17.7(718892)	UAC 15.3(622407)	UGC 12.6(513028)
UUA 7.7(311881)	UCA 12.2(496448)	UAA 1.0(40285)	UGA 1.6(63237)
UUG 12.9(525688)	UCG 4.4(179419)	UAG 0.8(32109)	UGG 13.2(535595)
CUU 13.2(536515)	CCU 17.5(713233)	CAU 10.9(441711)	CGU 4.5(184609)
CUC 19.6(796638)	CCC 19.8(804620)	CAC 15.1(613713)	CGC 10.4(423516)
CUA 7.2(290751)	CCA 16.9(688038)	CAA 12.3(501911)	CGA 6.2(250760)
CUG 39.6(1611801)	CCG 6.9(281570)	CAG 34.2(1391973)	CGG 11.4(464485)
AUU 16.0(650473)	ACU 13.1(533609)	AAU 17.0(689701)	AGU 12.1(493429)
AUC 20.8(846466)	ACC 18.9(768147)	AAC 19.1(776603)	AGC 19.5(791383)
AUA 7.5(304565)	ACA 15.1(614523)	AAA 24.4(993621)	AGA 12.2(494682)
AUG 22.0(896005)	ACG 6.1(246105)	AAG 31.9(1295568)	AGG 12.0(486463)
GUU 11.0(448607)	GCU 18.4(750096)	GAU 21.8(885429)	GGU 10.8(437126)
GUC 14.5(588138)	GCC 27.7(1127679)	GAC 25.1(1020595)	GGC 22.2(903565)
GUA 7.1(287712)	GCA 15.8(643471)	GAA 29.0(1177632)	GGA 16.5(669873)
GUG 28.1(1143534)	GCG 7.4(299495)	GAG 39.6(1609975)	GGG 16.5(669768)

The codon usage table of *Aedes aegypti*.

Aedes aegypti [gbinv]: 585 CDS's (257935 codons)

fields: [triplet] [frequency: per thousand] ([number])

UUU 11.8(3046)	UCU 8.8(2271)	UAU 11.2(2888)	UGU 8.8(2269)
UUC 30.5(7872)	UCC 15.7(4041)	UAC 23.7(6104)	UGC 12.3(3182)
UUA 5.0(1277)	UCA 8.8(2280)	UAA 1.1(271)	UGA 0.6(167)
UUG 18.9(4870)	UCG 18.5(4769)	UAG 0.6(150)	UGG 11.6(3003)
CUU 9.5(2455)	CCU 8.6(2217)	CAU 11.0(2846)	CGU 11.0(2826)
CUC 11.5(2957)	CCC 10.7(2757)	CAC 15.3(3951)	CGC 10.1(2597)
CUA 7.7(1977)	CCA 15.4(3978)	CAA 17.2(4442)	CGA 9.5(2440)
CUG 32.2(8317)	CCG 16.8(4327)	CAG 25.3(6522)	CGG 8.5(2183)
AUU 17.5(4522)	ACU 10.9(2817)	AAU 19.9(5140)	AGU 12.2(3144)
AUC 27.9(7193)	ACC 20.2(5209)	AAC 30.4(7848)	AGC 14.5(3728)
AUA 7.1(1828)	ACA 9.6(2469)	AAA 23.2(5988)	AGA 5.1(1323)
AUG 23.7(6104)	ACG 13.7(3538)	AAG 35.2(9088)	AGG 4.3(1107)
GUU 17.2(4435)	GCU 19.1(4939)	GAU 31.7(8183)	GGU 17.8(4603)
GUC 17.8(4600)	GCC 26.1(6721)	GAC 25.1(6466)	GGC 16.8(4334)
GUA 9.9(2549)	GCA 13.2(3409)	GAA 34.0(8758)	GGA 24.1(6212)
GUG 20.7(5332)	GCG 12.2(3135)	GAG 24.9(6414)	GGG 6.0(1547)
