# Supplementary material for: A small stretch of poor codon usage at the beginning of dengue virus open reading frame may act as a translational checkpoint
Source: BMC Res Notes. 2023 Dec 5;16:359. doi: 10.1186/s13104-023-06615-5 (PMC10698908; doi:10.1186/s13104-023-06615-5)
Supplement: Supplementary file 6 — Additional file 6: Table S6. Local CAI of DENV1-4 using codon usage table of Homo sapiens as a reference set [file 13104_2023_6615_MOESM6_ESM.pdf]

Table S8. RSCU values of checkpoint for DENV1-4.

Codon	AA	RSCU of CDS of complete genome				RSCU of checkpoint			
		DENV1	DENV2	DENV3	DENV4	DENV1 at 35-55 codons	DENV2 at 40-60 codons	DENV3 at 35-55 codons	DENV4 at 30-50 codons
GCA	A	1.37	1.57	1.28	1.18	1.60	1.38	0.76	4.00
GCC	A	1.30	1.13	1.14	1.31	0.80	1.16	0.98	0.00
GCG	A	0.38	0.31	0.48	0.36	0.00	0.55	0.84	0.00
GCU	A	0.95	0.99	1.10	1.15	1.60	0.91	1.42	0.00
UGC	C	0.95	0.99	0.97	1.05	0.00	0.00	0.00	0.00
UGU	C	1.05	1.01	1.03	0.95	0.00	0.00	0.00	0.00
GAC	D	1.13	1.18	1.12	1.13	0.00	0.00	0.00	0.00
GAU	D	0.87	0.82	0.88	0.87	0.00	0.00	0.00	0.00
GAA	E	1.21	1.40	1.17	1.27	0.00	0.00	0.00	0.00
GAG	E	0.79	0.60	0.83	0.73	0.00	0.00	0.00	0.00
UUC	F	1.05	1.07	0.95	0.82	1.22	1.95	1.43	1.18
UUU	F	0.95	0.93	1.05	1.18	0.78	0.00	0.58	0.82
GGA	G	2.33	2.25	2.10	2.03	2.63	2.63	3.12	2.04
GGC	G	0.52	0.56	0.64	0.50	1.19	0.00	0.82	0.00
GGG	G	0.66	0.73	0.84	0.92	0.04	1.37	0.00	1.96
GGU	G	0.48	0.46	0.42	0.55	0.14	0.00	0.06	0.00
CAC	H	1.11	0.98	1.11	0.99	0.00	0.00	0.00	0.00
CAU	H	0.89	1.02	0.89	1.01	0.00	0.00	0.00	0.00
AUA	I	1.37	1.18	1.31	1.18	2.03	0.76	1.48	0.03
AUC	I	0.82	1.02	0.74	0.91	0.03	2.00	0.16	1.97
AUU	I	0.82	0.80	0.95	0.91	0.95	0.24	1.36	1.01
AAA	K	1.34	1.27	1.17	1.24	1.00	1.70	0.99	1.68
AAG	K	0.66	0.73	0.83	0.76	1.00	0.30	1.01	0.32
CUA	L	1.28	1.05	0.92	0.89	1.99	1.61	0.97	0.77
CUC	L	0.66	0.92	0.90	1.02	0.48	0.03	0.92	0.20
CUG	L	1.48	1.50	1.20	1.34	0.38	1.68	1.52	1.20
CUU	L	0.69	0.64	0.84	0.66	0.55	1.36	0.08	1.79
UUA	L	0.72	0.73	0.81	0.74	0.03	0.81	0.00	0.99
UUG	L	1.17	1.16	1.32	1.35	2.58	0.53	2.52	1.05
AAC	N	1.09	1.02	1.14	1.16	0.00	0.00	1.60	0.00
AAU	N	0.91	0.98	0.86	0.84	0.00	0.00	0.25	0.00
CCA	P	2.31	2.31	2.19	1.87	1.33	3.57	3.67	2.32
CCC	P	0.75	0.68	0.75	1.01	2.37	0.00	0.03	1.16
CCG	P	0.33	0.27	0.19	0.36	0.00	0.43	0.27	0.32
CCU	P	0.62	0.74	0.88	0.76	0.30	0.00	0.03	0.21
CAA	Q	1.17	1.22	1.29	0.97	1.90	0.00	0.70	0.00
CAG	Q	0.83	0.78	0.71	1.03	0.10	0.00	1.30	0.00
AGA	R	3.15	3.39	3.31	3.06	6.00	2.05	5.93	3.04
AGG	R	1.36	1.20	1.55	1.76	0.00	0.00	0.08	0.10
CGA	R	0.50	0.45	0.31	0.45	0.00	1.15	0.00	1.18
CGC	R	0.42	0.39	0.34	0.32	0.00	0.70	0.00	0.00
CGG	R	0.28	0.17	0.24	0.22	0.00	0.05	0.00	1.68
CGU	R	0.30	0.40	0.26	0.18	0.00	2.05	0.00	0.00
AGC	S	0.84	0.98	0.89	0.79	0.90	0.00	0.00	0.00
AGU	S	0.73	0.95	0.72	0.75	0.00	0.00	0.00	0.04
UCA	S	2.21	2.02	2.15	2.06	5.05	6.00	0.15	1.78
UCC	S	1.04	0.85	0.96	1.00	0.00	0.00	0.00	3.26
UCG	S	0.28	0.37	0.45	0.43	0.05	0.00	0.00	0.20
UCU	S	0.90	0.82	0.84	0.97	0.00	0.00	0.00	0.72
ACA	T	1.80	2.04	2.12	1.78	4.00	3.33	2.05	1.39
ACC	T	0.94	0.79	0.74	1.04	0.00	0.03	1.95	1.11
ACG	T	0.53	0.49	0.50	0.50	0.00	0.63	0.00	1.40
ACU	T	0.72	0.68	0.64	0.68	0.00	0.00	0.00	0.11
GUA	V	0.59	0.57	0.58	0.66	0.00	0.00	0.00	0.00
GUC	V	0.80	1.00	0.90	0.89	0.00	0.05	1.60	1.42
GUG	V	1.76	1.62	1.66	1.69	4.00	3.95	0.10	2.00
GUU	V	0.85	0.81	0.85	0.76	0.00	0.00	2.30	0.58
UAC	Y	0.95	1.20	1.14	1.01	0.00	0.00	0.00	0.00
UAU	Y	1.05	0.80	0.86	0.99	0.00	0.00	0.00	0.00

Abbreviations: AA, amino acid; CDS, coding sequence.

Over-represented codons in CDS. (green)

Under-represented codons in CDS. (yellow)
