# Supplementary material for: A small stretch of poor codon usage at the beginning of dengue virus open reading frame may act as a translational checkpoint
Source: BMC Res Notes. 2023 Dec 5;16:359. doi: 10.1186/s13104-023-06615-5 (PMC10698908; doi:10.1186/s13104-023-06615-5)
Supplement: Supplementary file 7 — Additional file 7: Table S7. Local CAI of DENV1-4 using codon usage table of Aedes aegypti as a reference set [file 13104_2023_6615_MOESM7_ESM.pdf]

Table S1. Information of DENV complete genomes.

No.	Serotype	Accession No.	Length (bp)	Strain	Location	Year
1	DENV1	AF309641	10721	Dengue virus type 1 from Cambodia, complete genome	Cambodia	-
2	DENV1	AY762084	10733	Dengue virus type 1 strain Singapore 8114/93, complete genome	Singapore	-
3	DENV1	AY732483	10735	Dengue virus type 1 strain ThD1_0008_81, complete genome	Thailand	1981
4	DENV1	KR052012	10714	Dengue virus strain Hb33/CHN/2014, complete genome	China	2014
5	DENV1	JF459993	10735	Dengue virus 1 strain 49440 from Myanmar, complete genome	Myanmar	2002
6	DENV1	KM204119	10736	Dengue virus 1 strain Hawaii, complete genome	USA	1944
7	DENV1	DQ672559	10735	Dengue virus type 1 strain FP1104, complete genome	French Polynesia	-
8	DENV1	AY277666	10735	Dengue virus 1 isolate ARG0048, complete genome	Argentina	-
9	DENV1	HQ332183	10735	Dengue virus 1 strain VE_61081_2007, complete genome	Venezuela	2007
10	DENV1	KJ189304	10447	Dengue virus 1 isolate DENV-1/CO/BID-V7292/2005, complete genome	Colombia	2005
11	DENV1	JX669473	10736	Dengue virus 1 isolate 74488/BR-PE/01, complete genome	Brazil	2001
12	DENV1	KJ189369	10456	Dengue virus 1 isolate DENV-1/MX/BID-V8196/2011, complete genome	Mexico	2011
13	DENV1	KJ649286	10622	Dengue virus 1 isolate DENV-1-Jeddah, complete genome	Saudi Arabia	2011
14	DENV1	JF937608	10443	Dengue virus 1 isolate DENV-1/VN/BID-V3958/2008, complete genome	Viet Nam	2008
15	DENV1	EU081262	10735	Dengue virus type 1 strain D1/SG/05K4173DK1/2005, complete genome	Singapore	2005
16	DENV1	AY732482	10735	Dengue virus type 1 strain ThD1_0049_01, complete genome	Thailand	2001
17	DENV1	KF955446	10470	Dengue virus 1 isolate DENV-1/VN/BID-V3909/2008, complete genome	Viet Nam	2008
18	DENV1	DQ672563	10735	Dengue virus type 1 strain HawO3758, complete genome	USA	2001
19	DENV1	DQ672557	10735	Dengue virus type 1 strain FP0705, complete genome	French Polynesia	-
20	DENV1	KC762654	10735	Dengue virus 1 isolate MKS-0077, complete genom	Indonesia	2007
21	DENV1	OM281599	10736	Dengue virus 1 isolate 002/SH/2020, complete genome	Philippines	2020
22	DENV1	OM281598	10735	Dengue virus 1 isolate 144/SH/2019, complete genome	Thailand	2019
23	DENV1	OM281596	10735	Dengue virus 1 isolate 132/SH/2019, complete genome	Maldives	2019
24	DENV1	OR418422	10734	Dengue virus type 1 isolate 23GZ09930A_D1_F, complete genome	China	2023
25	DENV1	MG721063	10736	Dengue virus type 1 strain R1_J10, complete genome	India	2016
26	DENV1	ON123661	10735	Dengue virus type I isolate THSTI-TRC-DENV1-11, complete genome	India	2018
27	DENV1	OM281569	10735	Dengue virus 1 isolate 037/SH/2018, complete genome	Cambodia	2018
28	DENV1	MN018288	10735	Dengue virus type 1 isolate D16001, complete genome	Brazil	2016
29	DENV1	KY926848	10597	Dengue virus type 1 isolate PF13/190813-45, complete genome	French Polynesia	2013
30	DENV1	MH921567	10706	Dengue virus type 1 isolate PNG 2016, complete genome	Australia	2016
31	DENV1	KP398852	10735	Dengue virus 1 isolate DK87, complete genome	Sri Lanka	2014
32	DENV1	MG679801	10735	Dengue virus type 1 isolate DENV-1/China/MY22(2017), complete genome	Myanmar	2017
33	DENV1	MH450312	10752	Dengue virus type 1 isolate DENV1-VE-IDAMS-910132_2015-10-19, complete genome	Venezuela	2015
34	DENV1	KF973475	10615	Dengue virus 1 isolate DENV-1/NI/BID-V7696/2012, complete genome	Nicaragua	2012
35	DENV1	KJ189359	10444	Dengue virus 1 isolate DENV-1/PR/BID-V7719/2012, complete genome	Puerto Rico	2012
36	DENV1	KJ189368	10480	Dengue virus 1 isolate DENV-1/MX/BID-V8195/2012, complete genome	Mexico	2012
37	DENV1	KT279761	10735	Dengue virus type 1 strain Haiti/1207/2014, complete genome	Haiti	2014
38	DENV1	JN819417	10676	Dengue virus 1 isolate DENV-1/SV/BID-V2938/1993, complete genome	El Salvador	1993
39	DENV1	KU509249	10582	Dengue virus type 1 strain DENV1-17388, complete genome	Jamaica	2012
40	DENV1	MT076935	10735	Dengue virus type 1 isolate CFA348, complete genome	Kenya	2015
41	DENV2	KM204118	10723	Dengue virus 2 strain New Guinea C, complete genome	Papua New Guinea	1944
42	DENV2	EU359009	10723	Dengue virus 2 isolate ZH1340, complete genome	China	-
43	DENV2	M20558	10723	Dengue virus type 2 Jamaica/N.1409, complete genome	Jamaica	-
44	DENV2	AF208496	10722	Dengue virus type 2 strain DEN2/H/IMTSSA-MART/98-703, complete genome	Martinique	1998
45	DENV2	FJ744725	10678	Dengue virus 2 isolate DENV-2/TH/BID-V2311/2001, complete genome	Thailand	2001
46	DENV2	HQ332190	10722	Dengue virus 2 strain VE_61095_2007, complete genome	Venezuela	2007
47	DENV2	GQ868555	10668	Dengue virus 2 isolate DENV-2/CO/BID-V3371/2005, complete genome	Colombia	2005
48	DENV2	JX073928	10723	Dengue virus 2 strain BR DEN2 01-01, complete genome	Brazil	2001
49	DENV2	GU369819	10521	Dengue virus 2 isolate CAM7786, complete genome	Mexico	2002
50	DENV2	GQ398267	10723	Dengue virus 2 strain DENV-2/SG/07K3588DK1/2007, complete genome	Singapore	2007
51	DENV2	GQ398258	10723	Dengue virus 2 strain DENV-2/ID/1016DN/1975, complete genome	Indonesia	1975
52	DENV2	EU482672	10678	Dengue virus 2 isolate DENV-2/VN/BID-V735/2006, complete genome	Viet Nam	2006
53	DENV2	KF360005	10629	Dengue virus 2 isolate DENV-2/PK, complete genome	Pakistan	2010
54	DENV2	JF730050	10484	Dengue virus 2 isolate DENV-2/US/BID-V5412/2007, complete genome	USA	2007
55	DENV2	HM582110	10713	Dengue virus 2 strain D2/PF/UH00/1973, complete genome	French Polynesia	1973
56	DENV2	JF357907	10477	Dengue virus 2 isolate DENV-2/NI/BID-V4650/2007, complete genome	Nicaragua	2007
57	DENV2	EU056810	10723	Dengue virus type 2 isolate 1349, complete genome	Burkina Faso	1983
58	DENV2	EF457904	10724	Dengue virus type 2 isolate Dak Ar D75505, complete genome	Senegal	1999
59	DENV2	GU131843	10657	Dengue virus 2 isolate DENV-2/BF/BID-V3502/1986, complete genome	Burkina Faso	1986
60	DENV2	HQ999999	10725	Dengue virus 2 isolate DENV-2/GU/FDA-GUA09/2009, complete genome	Guatemala	2009
61	DENV2	OQ101603	10725	Dengue virus type 2 isolate C2_2019_DENV-2, complete genome	Brazil	2019
62	DENV2	MH985859	10722	Dengue virus type 2 isolate Van 2017, complete genome	Australia	2017
63	DENV2	MT180479	10723	Dengue virus type 2 isolate 231/17kandy, complete genome	Sri Lanka	2017
64	DENV2	MN018344	10724	Dengue virus type 2 isolate D17013, complete genome	Thailand	2017
65	DENV2	OQ652965	10710	Dengue virus type 2 isolate DENV2_YN_017, complete genome	Myanmar	2018
66	DENV2	MK411559	10676	Dengue virus type 2 isolate ID/JMB-001B/2016, complete genome	Indonesia	2016
67	DENV2	MG721054	10723	Dengue virus type 2 isolate R1_J17_DENV2, complete genome	India	2016
68	DENV2	MH069499	10712	Dengue virus type 2 strain DENV-2/VE/IDAMS/910105, complete genome	Venezuela	2015
69	DENV2	OQ782228	10724	Dengue virus type 2 isolate LLE8614, complete genome	Nicaragua	2018
70	DENV2	MH613986	10723	Dengue virus type 2 isolate C6L_P56, complete genome	Mexico	2017
71	DENV2	KX702403	10724	Dengue virus type 2 strain Dengue virus 2/Homo sapiens/Haiti-1/2016, complete genome	Haiti	2016
72	DENV2	KU509267	10567	Dengue virus type 2 strain DENV2-30, complete genome	Guatemala	2010
73	DENV2	EU920848	10724	Dengue virus 2 isolate FGU-Apr2-06, complete genome	French Guiana	2006

No.	Serotype	Accession No.	Length (bp)	Strain	Location	Year
74	DENV2	FJ898451	10678	Dengue virus 2 isolate DENV-2/DO/BID-V2955/2003, complete genome	Dominican Republic	2003
75	DENV2	OQ603290	10725	Dengue virus type 2 isolate 462966, complete genome	Colombia	2016
76	DENV2	KU517845	10723	Dengue virus type 2 isolate PG-CN10-13, complete genome	Papua New Guinea	2013
77	DENV2	MN018365	10724	Dengue virus type 2 isolate D151663, complete genome	Maldives	2015
78	DENV2	MG189962	10665	Dengue virus type 2 isolate D2_K2_RIJ_059/Dar es Salaam 2014, complete genome	Tanzania	2014
79	DENV2	MT076937	10723	Dengue virus type 2 isolate CFA298, complete genome	Kenya	2015
80	DENV2	KY627763	10675	Dengue virus type 2 strain 7754691/BF/2016, complete genome	Burkina Faso	2016
81	DENV3	EF629370	10696	Dengue virus type 3 strain BR DEN3 RO1-02, complete genome	Brazil	-
82	DENV3	HQ332171	10707	Dengue virus 3 strain VE_61035_2006, complete genome	Venezuela	2006
83	DENV3	GU131954	10525	Dengue virus 3 isolate DENV-3/CO/BID-V3404/2006, complete genome	Colombia	2006
84	DENV3	FJ898442	10663	Dengue virus 3 isolate DENV-3/MX/BID-V2989/2007, complete genome	Mexico	2007
85	DENV3	KF041259	10675	Dengue virus 3 isolate D3/Pakistan/43298/2006, complete genome	Pakistan	2006
86	DENV3	AY766104	10696	Dengue virus type 3 strain Singapore 8120/95, complete genome	Singapore	-
87	DENV3	AY496871	10707	Dengue virus type 3 isolate BDH02-1, complete genome	Bangladesh	2002
88	DENV3	KF955460	10640	Dengue virus 3 isolate DENV-3/VN/BID-V1933/2008, complete genome	Viet Nam	2008
89	DENV3	FJ850056	10649	Dengue virus 3 isolate DENV-3/US/BID-V1611/2004, complete genome	USA	2004
90	DENV3	AY744679	10707	Dengue virus type 3 isolate PF90/3050, complete genome	French Polynesia	1990
91	DENV3	KF973487	10569	Dengue virus 3 isolate DENV-3/NI/BID-V7699/2011, complete genome	Nicaragua	2011
92	DENV3	AY676353	10707	Dengue virus type 3 strain ThD3_0007_87, complete genome	Thailand	1987
93	DENV3	AY923865	10707	Dengue virus type 3 strain C0360/94, complete genome	Indonesia	1994
94	DENV3	KU050695	10696	Dengue virus 3, complete genome	Philippines	1956
95	DENV3	AY099337	10707	Dengue virus type 3 isolate D3/H/IMTSSA-MART/1999/1243, complete genome	Martinique	1999
96	DENV3	KT726361	10663	Dengue virus 3 isolate Cuba_20_2002, complete genome	Cuba	2002
97	DENV3	KF955468	10640	Dengue virus 3 isolate DENV-3/PR/BID-V2116/2001, complete genome	Puerto Rico	2001
98	DENV3	FJ882575	10663	Dengue virus 3 isolate DENV-3/MZ/BID-V2418/1985, complete genome	Mozambique	1985
99	DENV3	FJ898456	10663	Dengue virus 3 isolate DENV-3/WS/BID-V2973/1995, complete genome	Samoa	1995
100	DENV3	FJ644564	10707	Dengue virus 3 isolate ND143 from India, complete genome	India	2007
101	DENV3	KF955473	10459	Dengue virus 3 isolate DENV-3/BR/BID-V2383/2002, complete genome	Brazil	2002
102	DENV3	KU509283	10336	Dengue virus type 3 strain DENV3-3404, complete genome	Sri Lanka	2006
103	DENV3	OR418423	10700	Dengue virus type 3 isolate 23GZ10604_D1_THA_F, complete genome	Thailand	2023
104	DENV3	MH823209	10707	Dengue virus type 3 isolate SMD-031, complete genome	Indonesia	2016
105	DENV3	MZ285732	10735	Dengue virus type 3 isolate dev1, complete genome	India	2019
106	DENV3	MW396468	10707	Dengue virus type 3 isolate IN043, complete genome	Bangladesh	2019
107	DENV3	MN018389	10708	Dengue virus type 3 isolate D17011, complete genome	Philippines	2017
108	DENV3	MN018381	10708	Dengue virus type 3 isolate D16016, complete genome	Malaysia	2016
109	DENV3	KJ830751	10635	Dengue virus 3 isolate Jeddah-2014, complete genome	Saudi Arabia	2014
110	DENV3	GU131878	10638	Dengue virus 3 isolate DENV-3/BR/BID-V3615/2007, complete genome	Brazil	2007
111	DENV3	FJ898459	10663	Dengue virus 3 isolate DENV-3/TT/BID-V2982/2002, complete genome	Trinidad and Tobago	2002
112	DENV3	KF955456	10645	Dengue virus 3 isolate DENV-3/PR/BID-V1728/2006, complete genome	Puerto Rico	2006
113	DENV3	KJ189301	10693	Dengue virus 3 isolate DENV-3/PE/BID-V7289/2008, complete genome	Peru	2008
114	DENV3	OK605762	10707	Dengue virus type 3 isolate DENV3-1_FPA0099, complete genome	Paraguay	2007
115	DENV3	KF973486	10576	Dengue virus 3 isolate DENV-3/NI/BID-V7694/2012, complete genome	Nicaragua	2012
116	DENV3	MH544651	10706	Dengue virus type 3 isolate 476947_Risaralda_CO_2016, complete genome	Colombia	2016
117	DENV3	MH888333	10683	Dengue virus type 3 isolate BOL_4025, complete genome	Bolivia	2011
118	DENV3	KU509278	10270	Dengue virus type 3 strain DENV3-254, complete genome	Barbados	2007
119	DENV3	KY794786	10643	Dengue virus type 3 isolate 101027, complete genome	Papua New Guinea	2010
120	DENV3	OK605766	10707	Dengue virus type 3 isolate DENV3-5_S_162, complete genome	Somalia	1993
121	DENV4	AY762085	10649	Dengue virus type 4 strain Singapore 8976/95, complete genome	Singapore	-
122	DENV4	HQ332176	10649	Dengue virus 4 strain VE_61054_2007, complete genome	Venezuela	2007
123	DENV4	FJ024476	10606	Dengue virus 4 isolate DENV-4/CO/BID-V1600/1997, complete genome	Colombia	1997
124	DENV4	JN983813	10649	Dengue virus 4 isolate Br246RR/10, complete genom	Brazil	2010
125	DENV4	KC333651	10574	Dengue virus 4 strain GZ/9809/2012, complete genome	China	2012
126	DENV4	KF041260	10652	Dengue virus 4 isolate D4/Pakistan/150/2009, complete genome	Pakistan	2009
127	DENV4	GQ252675	10591	Dengue virus 4 isolate DENV-4/US/BID-V2432/1995, complete genome	USA	1995
128	DENV4	AY618993	10653	Dengue virus type 4 strain ThD4_0734_00, complete genome	Thailand	2000
129	DENV4	KU523872	10653	Dengue virus 4 strain ID-CN27-15, complete genome	Indonesia	2015
130	DENV4	KU523871	10589	Dengue virus 4 strain PH-CN08-14, complete genome	Philippines	2014
131	DENV4	JF262783	10659	Dengue virus 4 isolate INDIA G11337, complete genome	India	1961
132	DENV4	KJ160504	10650	Dengue virus 4 isolate rDENV4, complete genome	Sri Lanka	-
133	DENV4	KT794007	10649	Dengue virus 4 isolate BR005AM_2011, complete genome	Brazil	2011
134	DENV4	EF457906	10666	Dengue virus type 4 isolate P75-215, complete genome	Malaysia	1975
135	DENV4	AY618989	10653	Dengue virus type 4 strain ThD4_0017_97, complete genome	Thailand	1997
136	DENV4	KC762699	10641	Dengue virus 4 isolate MKS-2139, complete genome	Indonesia	2008
137	DENV4	FJ639764	10566	Dengue virus 4 isolate DENV-4/VE/BID-V2194/2001, complete genome	Venezuela	2001
138	DENV4	GQ868585	10606	Dengue virus 4 isolate DENV-4/CO/BID-V3412/2005, complete genome	Colombia	2005
139	DENV4	JF262782	10649	Dengue virus 4 isolate Haiti73, complete genome	Haiti	1994
140	DENV4	AF326573	10649	Dengue virus type 4 strain 814669, complete genome	Dominica	1981
141	DENV4	KU513441	10650	Dengue virus type 4 isolate LRV13/422, complete genome	Brazil	2013
142	DENV4	MW793460	10648	Dengue virus type 4 isolate 1036, complete genome	Thailand	2021
143	DENV4	OL314747	10653	Dengue virus 4 isolate JBB-055, complete genome	Indonesia	2019
144	DENV4	ON799403	10649	Dengue virus type 4 isolate THSTI-TRC-DENV4-02, complete genome	India	2018
145	DENV4	MN018394	10652	Dengue virus type 4 isolate D151435, complete genome	Cambodia	2015
146	DENV4	MH888334	10605	Dengue virus type 4 isolate CNR_16861, complete genome	Malaysia	2013
147	DENV4	MH382789	10653	Dengue virus type 4 isolate PNG 2016a, complete genome	Australia	2016
148	DENV4	MN018395	10652	Dengue virus type 4 isolate D151453, complete genome	Philippines	2015

No.	Serotype	Accession No.	Length (bp)	Strain	Location	Year
149	DENV4	MN018393	10652	Dengue virus type 4 isolate D15312, complete genome	Philippines	2015
150	DENV4	MN018398	10652	Dengue virus type 4 isolate D16039, complete genome	China	2016
151	DENV4	JN638571	10656	Dengue virus 4 isolate DHF patient, complete genome	Cambodia	2007
152	DENV4	OP411002	10653	Dengue virus type 4 isolate SG(EHI)D4/30932Y19, complete genome	Singapore	2019
153	DENV4	ON799402	10649	Dengue virus type 4 isolate THSTI-TRC-DENV4-01, complete genome	India	2020
154	DENV4	MN018396	10652	Dengue virus type 4 isolate D151602, complete genome	Thailand	2015
155	DENV4	MN018397	10652	Dengue virus type 4 isolate D16004, complete genome	Viet Nam	2016
156	DENV4	MN018390	10652	Dengue virus type 4 isolate D13467, complete genome	China	2013
157	DENV4	AY776330	10353	Dengue virus type 4 strain Taiwan-2K0713 polyprotein gene, complete cds	Taiwan	-
158	DENV4	KY670635	10617	Dengue virus type 4 strain D4/Philippines/0310aTw, complete genome	Taiwan	2003
159	DENV4	MK514144	10649	Dengue virus type 4 isolate Homo sapiens/Haiti-0075/2015, complete genome	Haiti	2015
160	DENV4	MT076955	10648	Dengue virus type 4 isolate KFA236, complete genome	Kenya	2015
