# Supplementary material for: A small stretch of poor codon usage at the beginning of dengue virus open reading frame may act as a translational checkpoint
Source: BMC Res Notes. 2023 Dec 5;16:359. doi: 10.1186/s13104-023-06615-5 (PMC10698908; doi:10.1186/s13104-023-06615-5)
Supplement: Supplementary file 8 — Additional file 8: Table S8. RSCU values of checkpoint for DENV1-4 [file 13104_2023_6615_MOESM8_ESM.pdf]

Table S3. RSCU values of DENV1-4 in the CDS of the entire genome and their various initiation sites; the first 25, 50, 75, and 100 codons; and the human host.

Codon	AA	RSCU of CDS of complete genome				RSCU of *Homo sapiens*	RSCU of the first 25 codons				RSCU of the first 50 codons				RSCU of the first 75 codons				RSCU of first 100 codons			
		DENV1	DENV2	DENV3	DENV4		DENV1	DENV2	DENV3	DENV4	DENV1	DENV2	DENV3	DENV4	DENV1	DENV2	DENV3	DENV4	DENV1	DENV2	DENV3	DENV4
GCA	A	1.37	1.57	1.28	1.18	0.91	0.00	0.00	0.00	0.00	0.00	0.00	0.00	3.79	1.09	1.38	0.63	2.61	1.00	1.11	0.54	2.60
GCC	A	1.30	1.13	1.14	1.31	1.60	0.00	0.00	0.00	0.00	0.00	1.95	0.00	0.00	0.55	0.98	0.81	1.26	0.48	0.93	1.10	1.25
GCG	A	0.38	0.31	0.48	0.36	0.43	3.30	4.00	0.10	0.00	2.43	2.00	3.97	0.00	1.09	1.56	1.38	0.00	1.40	1.25	1.19	0.00
GCU	A	0.95	0.99	1.10	1.15	1.06	0.70	0.00	0.00	0.40	1.57	0.05	0.03	0.21	1.27	0.08	1.18	0.13	1.12	0.73	1.17	0.16
UGC	C	0.95	0.99	0.97	1.05	1.09	0.00	0.00	0.00	0.00	0.00	0.00	0.00	0.00	0.00	0.00	0.00	0.00	0.00	0.00	0.00	0.00
UGU	C	1.05	1.01	1.03	0.95	0.91	0.00	0.00	0.00	0.00	0.00	0.00	0.00	0.00	0.00	0.00	0.00	0.00	0.00	0.00	0.00	0.00
GAC	D	1.13	1.18	1.12	1.13	1.07	0.00	0.00	0.00	0.00	0.00	0.00	0.00	0.00	0.00	0.00	0.00	0.00	0.00	0.00	0.00	0.00
GAU	D	0.87	0.82	0.88	0.87	0.93	0.00	0.00	0.00	0.00	0.00	0.00	0.00	0.00	0.00	0.00	0.00	0.00	0.00	0.00	0.00	0.00
GAA	E	1.21	1.40	1.17	1.27	0.84	0.00	0.00	0.00	0.00	0.00	0.00	0.00	0.00	0.00	0.00	0.00	0.00	1.10	0.03	0.05	0.03
GAG	E	0.79	0.60	0.83	0.73	1.15	0.00	2.00	0.00	2.00	0.00	2.00	0.00	2.00	0.00	2.00	0.00	2.00	0.85	1.98	1.95	1.97
UUC	F	1.05	1.07	0.95	0.82	1.07	2.00	2.00	0.05	2.00	1.80	1.97	1.93	1.65	1.48	1.97	1.54	1.35	1.54	1.94	1.61	1.44
UUU	F	0.95	0.93	1.05	1.18	0.93	0.00	0.00	0.00	0.00	0.20	0.03	0.07	0.35	0.52	0.03	0.46	0.65	0.46	0.06	0.39	0.56
GGA	G	2.33	2.25	2.10	2.03	1.00	0.10	0.00	3.90	0.00	2.10	3.90	3.17	2.05	2.42	3.14	2.94	2.02	2.11	2.84	2.62	1.96
GGC	G	0.52	0.56	0.64	0.50	1.34	0.05	0.00	0.00	0.00	0.96	0.00	0.81	0.00	0.86	0.00	0.52	0.00	0.76	0.04	0.88	0.30
GGG	G	0.66	0.73	0.84	0.92	1.00	0.00	0.00	0.00	0.00	0.00	0.10	0.00	1.95	0.03	0.86	0.49	1.98	0.03	1.11	0.43	1.58
GGU	G	0.48	0.46	0.42	0.55	0.65	2.45	0.00	0.10	0.00	0.95	0.00	0.03	0.00	0.68	0.00	0.05	0.00	1.11	0.00	0.07	0.17
CAC	H	1.11	0.98	1.11	0.99	1.16	0.00	0.00	0.00	0.00	0.00	0.00	0.00	0.00	0.00	0.00	0.00	0.00	0.00	0.00	0.00	0.00
CAU	H	0.89	1.02	0.89	1.01	0.84	0.00	0.00	0.00	0.00	0.00	0.00	0.00	0.00	0.00	0.00	0.00	0.00	0.00	0.00	0.00	0.00
AUA	I	1.37	1.18	1.31	1.18	0.51	0.00	0.08	0.00	0.00	2.96	0.08	1.54	0.00	2.01	1.03	1.01	0.03	1.48	0.51	0.74	0.84
AUC	I	0.82	1.02	0.74	0.91	1.41	0.08	0.00	2.93	0.00	0.04	0.00	1.46	2.88	0.04	1.80	1.07	1.97	0.93	1.83	1.35	1.45
AUU	I	0.82	0.80	0.95	0.91	1.08	0.00	0.00	0.00	0.00	0.00	0.00	0.00	0.12	0.94	0.18	0.93	1.01	0.58	0.66	0.91	0.72
AAA	K	1.34	1.27	1.17	1.24	0.87	1.25	1.34	1.18	1.34	1.29	1.19	1.20	1.19	0.97	1.39	0.92	1.03	1.21	1.45	0.88	0.95
AAG	K	0.66	0.73	0.83	0.76	1.13	0.75	0.66	0.82	0.66	0.71	0.81	0.80	0.81	1.03	0.61	1.08	0.97	0.79	0.55	1.12	1.05
CUA	L	1.28	1.05	0.92	0.89	0.43	0.00	0.00	0.00	0.00	0.00	0.34	0.03	0.89	1.49	1.08	0.73	0.54	1.43	0.84	0.66	0.44
CUC	L	0.66	0.92	0.90	1.02	1.17	0.00	0.00	0.00	0.00	0.56	0.02	0.00	0.23	0.36	0.03	0.69	0.13	0.29	0.02	0.55	0.10
CUG	L	1.48	1.50	1.20	1.34	2.37	6.00	6.00	6.00	5.93	1.64	3.51	2.91	1.30	1.03	2.62	1.89	1.46	0.92	2.53	2.56	1.86
CUU	L	0.69	0.64	0.84	0.66	0.79	0.00	0.00	0.00	0.08	0.66	1.16	0.03	1.01	0.41	1.40	0.06	1.21	0.33	1.08	0.05	0.91
UUA	L	0.72	0.73	0.81	0.74	0.46	0.00	0.00	0.00	0.00	0.00	0.45	0.00	1.14	0.02	0.50	0.00	0.73	0.40	0.39	0.05	0.72
UUG	L	1.17	1.16	1.32	1.35	0.77	0.00	0.00	0.00	0.00	3.15	0.51	3.03	1.43	2.69	0.38	2.64	1.93	2.63	1.13	2.14	1.96
AAC	N	1.09	1.02	1.14	1.16	1.06	1.50	0.93	1.50	1.33	1.50	0.93	1.54	1.33	1.23	0.93	1.54	1.15	1.26	1.03	1.63	1.25
AAU	N	0.91	0.98	0.86	0.84	0.94	0.50	1.07	0.50	0.67	0.50	1.07	0.46	0.67	0.78	1.07	0.47	0.85	0.74	0.97	0.37	0.75
CCA	P	2.31	2.31	2.19	1.87	1.11	0.00	0.00	0.00	1.28	0.00	1.95	1.95	0.96	1.00	2.68	2.75	1.80	1.00	2.68	2.75	1.80
CCC	P	0.75	0.68	0.75	1.01	1.30	0.00	0.00	0.00	0.07	1.95	0.00	0.05	0.93	1.78	0.00	0.03	0.62	1.78	0.00	0.03	0.62
CCG	P	0.33	0.27	0.19	0.36	0.45	4.00	0.00	4.00	0.07	2.00	0.05	2.00	0.05	1.00	0.33	1.20	0.19	1.00	0.33	1.20	0.19
CCU	P	0.62	0.74	0.88	0.76	1.14	0.00	4.00	0.00	2.58	0.05	2.00	0.00	2.06	0.23	1.00	0.03	1.39	0.23	1.00	0.03	1.39
CAA	Q	1.17	1.22	1.29	0.97	0.53	2.00	2.00	2.00	2.00	1.30	1.11	0.90	2.00	1.30	1.11	0.90	1.35	1.30	1.11	0.90	1.35
CAG	Q	0.83	0.78	0.71	1.03	1.46	0.00	0.00	0.00	0.00	0.70	0.89	1.10	0.00	0.70	0.89	1.10	0.65	0.70	0.89	1.10	0.65
AGA	R	3.15	3.39	3.31	3.06	1.29	1.20	2.36	1.49	2.37	2.00	2.56	2.63	2.54	3.00	2.66	3.53	2.68	2.84	2.50	3.16	2.27
AGG	R	1.36	1.20	1.55	1.76	1.27	0.00	0.00	0.00	0.03	0.00	0.00	0.00	0.04	0.00	0.00	0.02	0.06	0.66	1.25	0.17	1.16
CGA	R	0.50	0.45	0.31	0.45	0.66	1.50	0.61	0.14	1.11	1.25	0.93	0.10	0.79	0.94	0.72	0.08	1.13	0.62	0.40	0.06	0.75
CGC	R	0.42	0.39	0.34	0.32	1.10	2.37	2.43	1.52	2.41	1.98	2.03	1.14	1.71	1.48	1.58	0.83	1.32	0.99	1.20	0.70	1.26
CGG	R	0.28	0.17	0.24	0.22	1.21	0.90	0.61	1.38	0.09	0.75	0.45	1.04	0.92	0.56	0.35	0.76	0.80	0.88	0.20	1.24	0.53
CGU	R	0.30	0.40	0.26	0.18	0.48	0.03	0.00	1.46	0.00	0.03	0.02	1.09	0.00	0.02	0.69	0.79	0.00	0.01	0.44	0.67	0.05
AGC	S	0.84	0.98	0.89	0.79	1.44	0.00	0.00	0.00	0.00	0.00	0.00	0.00	0.00	0.39	0.00	0.00	0.00	0.57	0.00	0.79	0.00
AGU	S	0.73	0.95	0.72	0.75	0.90	0.00	1.20	0.00	0.00	0.00	0.80	0.00	0.00	0.09	0.60	0.00	0.03	0.07	0.64	0.04	0.03
UCA	S	2.21	2.02	2.15	2.06	0.90	3.00	3.23	3.00	5.55	4.77	4.40	4.47	3.61	4.59	4.86	3.73	2.70	3.99	4.80	3.52	3.32
UCC	S	1.04	0.85	0.96	1.00	1.31	0.00	0.00	0.00	0.00	0.00	0.00	0.04	1.30	0.00	0.00	0.03	2.46	0.30	0.00	0.02	1.97
UCG	S	0.28	0.37	0.45	0.43	0.32	0.00	1.58	0.00	0.30	0.03	0.80	0.00	0.32	0.02	0.54	1.05	0.23	0.03	0.53	0.75	0.22
UCU	S	0.90	0.82	0.84	0.97	1.12	3.00	0.00	3.00	0.15	1.20	0.00	1.49	0.78	0.92	0.00	1.20	0.58	1.04	0.04	0.87	0.47
ACA	T	1.80	2.04	2.12	1.78	1.14	0.00	0.05	0.00	0.00	0.00	1.30	0.00	0.04	1.33	2.32	1.01	1.02	1.33	2.25	1.59	1.21
ACC	T	0.94	0.79	0.74	1.04	1.42	0.00	0.00	0.00	4.00	0.00	0.00	0.00	2.47	0.00	0.02	0.98	1.88	0.00	0.03	0.78	1.45
ACG	T	0.53	0.49	0.50	0.50	0.46	2.00	2.00	2.00	0.00	2.00	1.40	2.00	1.39	1.33	1.01	1.01	1.03	1.33	0.87	0.81	1.13
ACU	T	0.72	0.68	0.64	0.68	0.98	2.00	1.95	2.00	0.00	2.00	1.30	2.00	0.11	1.33	0.65	1.01	0.08	1.33	0.86	0.81	0.22
GUA	V	0.59	0.57	0.58	0.66	0.47	0.05	0.30	0.00	1.40	0.03	0.20	0.00	0.83	0.03	0.20	0.00	0.69	0.05	0.18	0.00	0.67
GUC	V	0.80	1.00	0.90	0.89	0.96	0.00	0.00	0.00	0.30	0.00	0.00	0.23	0.20	0.00	0.03	0.80	0.65	0.00	0.44	1.44	0.72
GUG	V	1.76	1.62	1.66	1.69	1.85	2.80	3.70	3.95	1.40	3.20	3.80	2.67	2.44	3.20	3.78	2.07	2.02	3.38	2.82	1.67	1.97
GUU	V	0.85	0.81	0.85	0.76	0.72	1.15	0.00	0.05	0.90	0.77	0.00	1.10	0.53	0.77	0.00	1.13	0.64	0.58	0.56	0.90	0.63
UAC	Y	0.95	1.20	1.14	1.01	1.11	0.00	0.00	0.00	0.00	0.00	0.00	0.00	0.00	0.00	0.00	0.00	0.00	0.05	0.00	0.00	0.00
UAU	Y	1.05	0.80	0.86	0.99	0.89	0.00	0.00	0.00	0.00	0.00	0.00	0.00	0.00	0.00	0.00	0.00	0.00	0.00	0.00	0.00	0.00

Abbreviations: AA, amino acid; CDS, coding sequence.

Over-represented codons in CDS.

Under-represented codons in CDS.
